# Usability of Telemedicine in Relation to Acceptability, Psychosocial Impact, and Future Use

**DOI:** 10.1093/geroni/igaa057.3408

**Published:** 2020-12-16

**Authors:** Katja Prevodnik, Simona Hvalič-Touzery, Vesna Dolničar, Maja Škafar, Andraž Petrovčič

**Affiliations:** University of Ljubljana, Ljubljana, Slovenia

## Abstract

This poster presents the results of an intervention study exploring how engagement in telemedicine at home affects chronic patients’ perceptions of usability and acceptability of the employed equipment, perceptions of its psychosocial impact, and intention of future use in the context of population aging. A purposively selected sample of 103 patients (mean age: 58 years) with chronic conditions (diabetes and/or hypertension) recruited in a community health center in Slovenia tested a home telemedicine system (TMS). After three months of utilization, an assessment of the relative importance of the usability and acceptance of TMS as factors influencing the patients’ self-reported psychosocial perception of TMS and intention of future use was performed based on a proposed structural equation model explaining these interdependencies. The results confirmed four of eight tested hypotheses. Notably, the intensity of TMS use was found to affect the evaluation of its usability, the perception of its psychosocial impact, and the intent of future use. Usability was found to be the main factor directly influencing acceptability, perception of psychosocial impact and intent for future use, whereas acceptability did not significantly affect either the perception of the psychosocial impact of TMS or the intent of future use.

